# Supplementary material for: Spatial continuity of neurons explains non-random network architecture
Source: iScience. 2026 Jun 1;29(6):116144. doi: 10.1016/j.isci.2026.116144 (PMC13253143; doi:10.1016/j.isci.2026.116144)
Supplement: Document S1. Figures S1–S12 and Methods S1 and S2 [file mmc1.pdf]

iScience, Volume *29*

## Supplemental information

# Spatial continuity of neurons explains non-random network architecture

**Michael W. Reimann, Daniela Egas Santander, Lida Kanari, and Natalí Barros-Zulaica**

# 1 Supplementary Figures

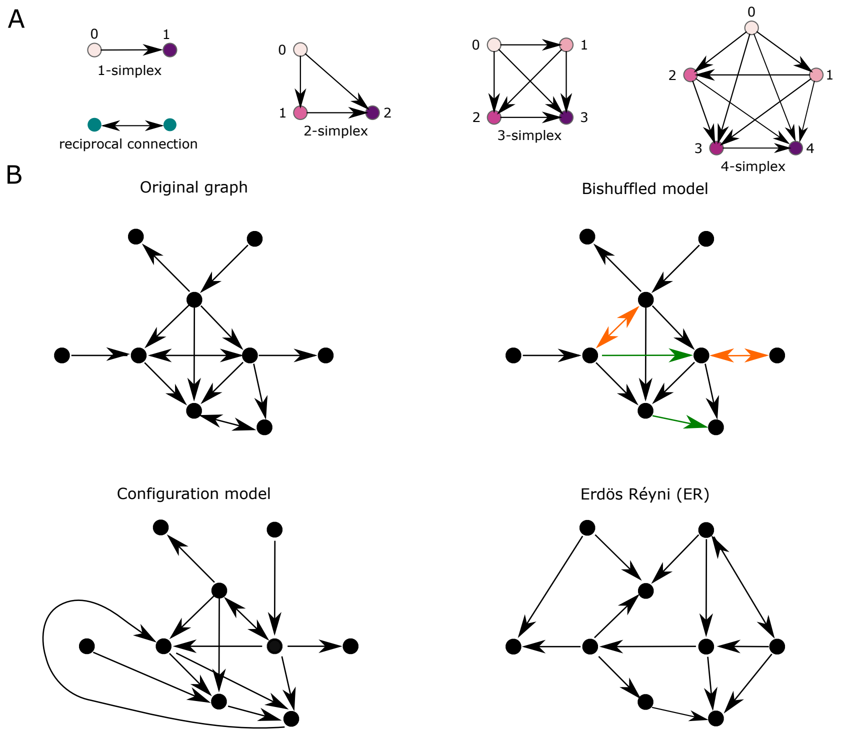


Figure S1: **Simplex motifs and stochastic controls.** Related to STAR Methods. A: Simplex motifs for dimensions 1 through 5. These are fully connected feedforward motifs in which all nodes are connected such that there exists an ordering where there is an edge from $v$ to $u$ whenever $v < u$. In the diagram, this ordering is both labeled and reflected in the color scheme, transitioning from lighter to darker hues. B: Different stochastic controls of an given original graph (top left). The bishuffled control alters only the positions of reciprocal (bidirectional) connections. Green edges indicate those that were reciprocal in the original, red edges indicate those that become reciprocal in the control. The configuration model and Erdős–Rényi (ER) models generate random graphs with the same number of edges as the original. The configuration model additionally preserves the in-degree and out-degree of each node, while ER scatters them at random across all edges.

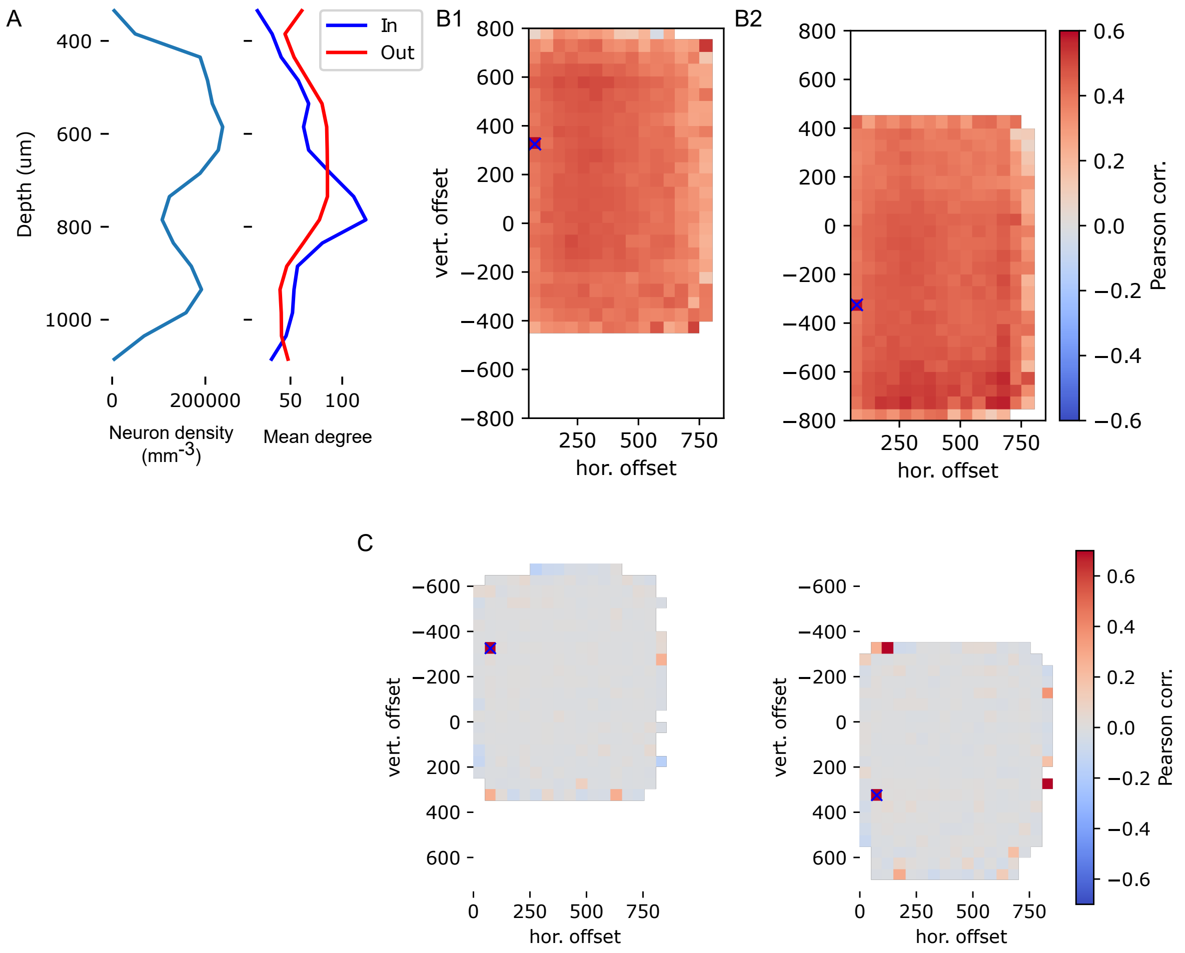


Figure S2: **Results for a configuration model with randomized soma locations.** Related to Figure 4. A, left: Neuron density against depth in the part of the MICrONS data we considered in this work. A, right: Mean in- and out-degree of neurons in depth bins in the MICrONS network we considered in this work. B: As Fig. 4B, but for a configuration model control with neuron locations randomized to uniform density. C: As B, but after normalization against out-degree. That is, as Fig. 4C, but for the configuration model control. Note that the normalization almost completely erases the correlations.

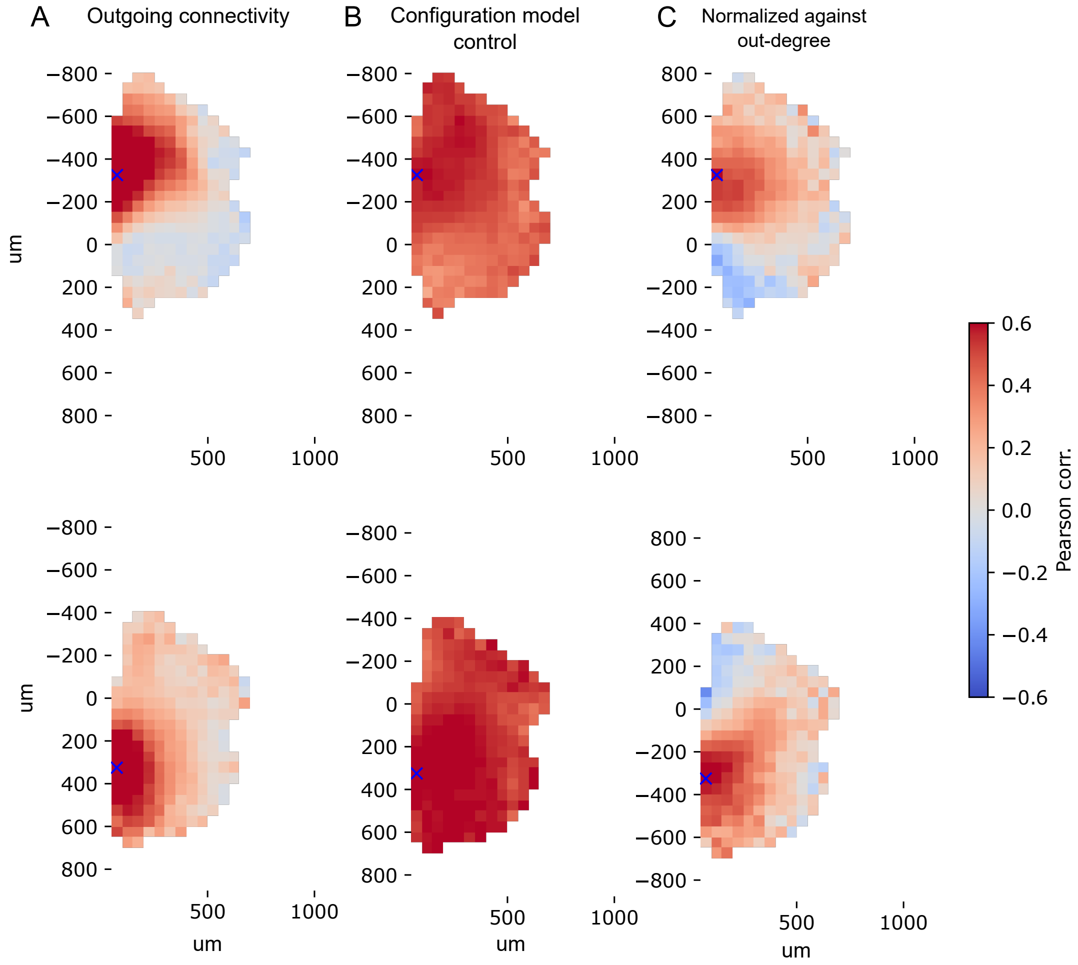


Figure S3: **Spatial correlations for an apposition-based connectome**. Related to Figure 4. Pearson correlations of connection probabilities into spatial bins as in Fig. 4, but for the apposition-based potential connectome instead of the electron-microscopically measured one.

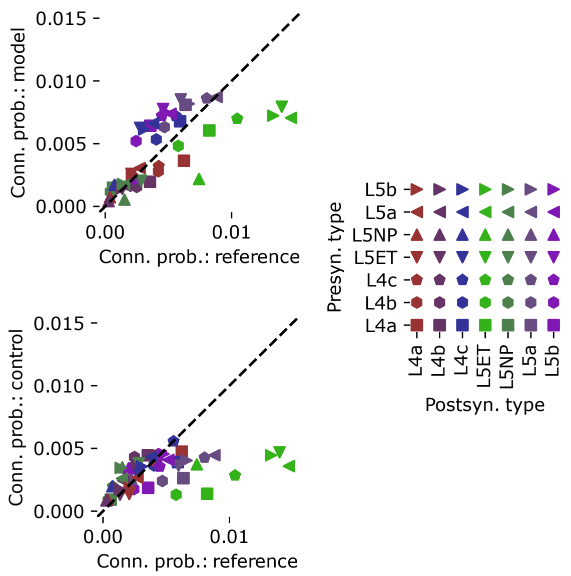


Figure S4: **Morphology type-specific pathways in model and data.** Related to Figure 5. Pathway-specific connection probabilities, reference (MICrONS) against SGSG model (top, pearsonr=0.7) and against distance-dependent control (bottom, pearsonr=0.45). Mean over five instances. Marker shape indicates pre-synaptic, color post-synaptic neuron types.

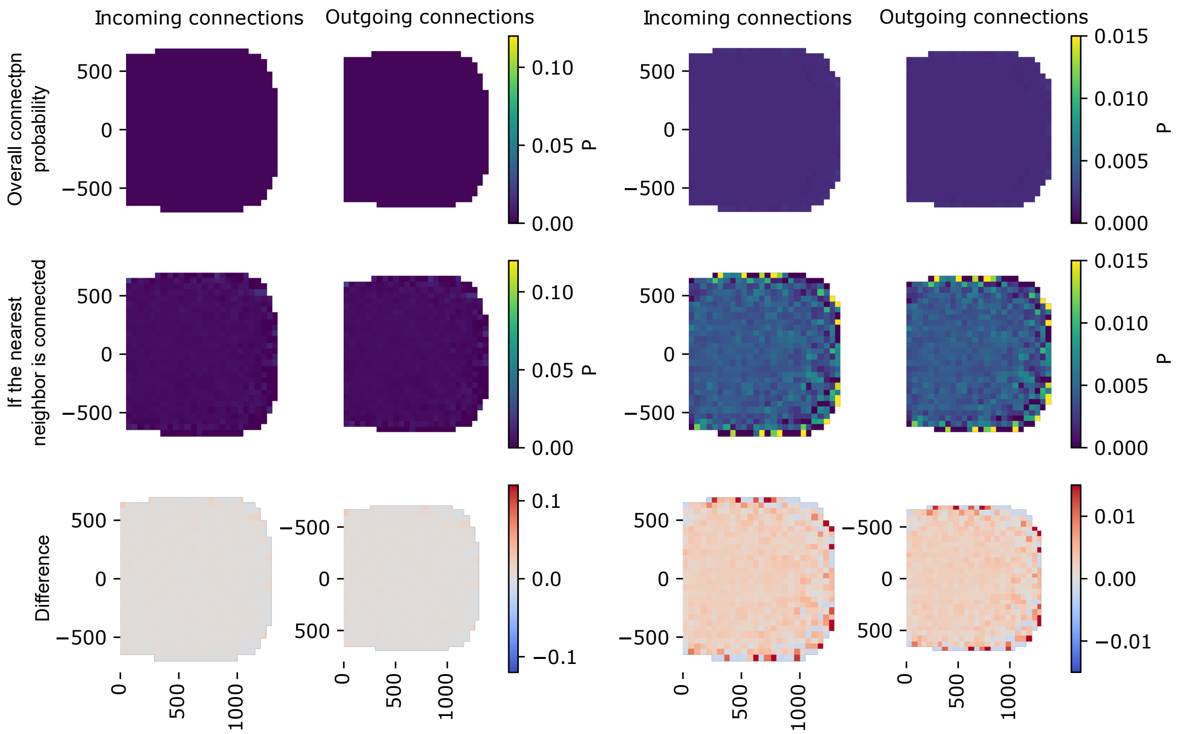


Figure S5: **Nearest-neighbor connection probability effect in a preferential attachment model.** Related to Figure 2. Incoming and outgoing connection probabilities as in Fig. 2 for a preferential attachment model fit to the MICrONS data. Top: Overall connection probability. Middle: Connection probability if the nearest neighbor is connected. Bottom: Difference. Left: Same colormap as in Fig. 2; right: colormap re-scaled.

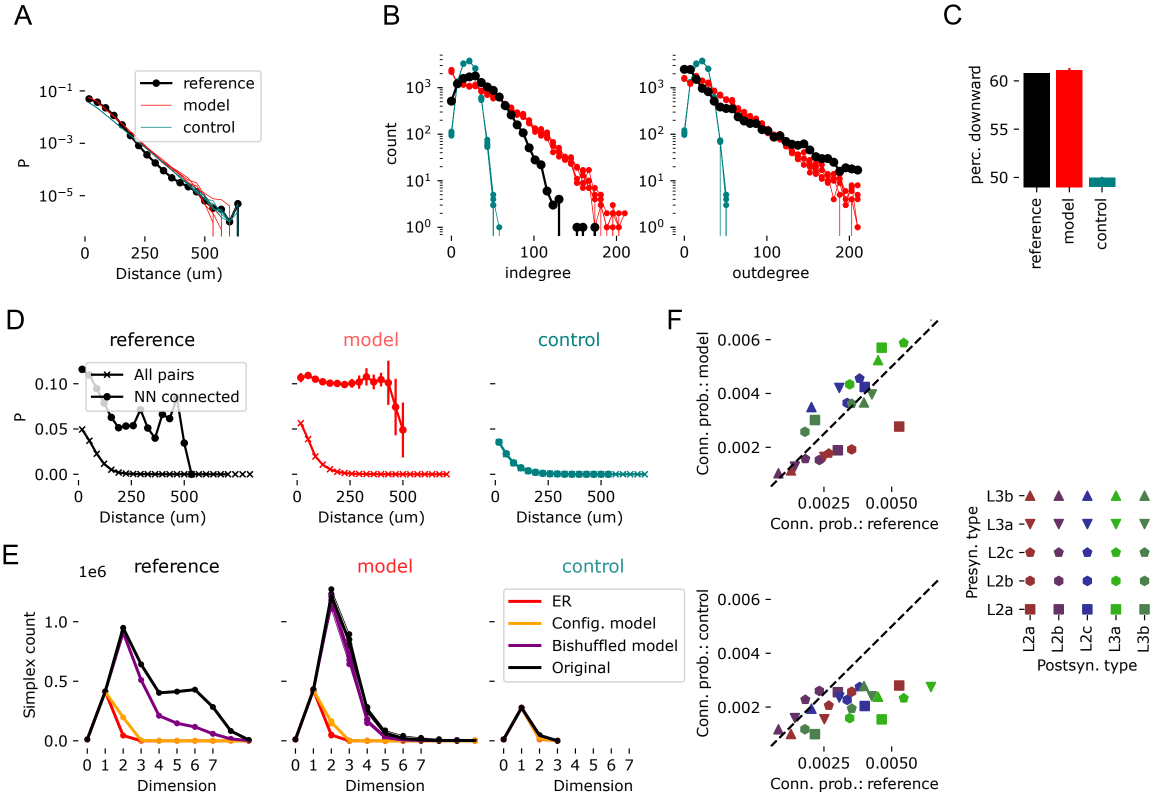


Figure S6: **Modeling connectivity in layers 2/3 of electron-microscopy data.** Related to Figure 5. SGSG model compared to excitatory connectivity in layers 2/3 of the MICrONS data. A-E: as in Figure 5, but for layers 2/3 instead of 4/5. F: As in Fig S4. Note that as a test of generalization, parameters $d$ and $p$ were not re-fitted and $q$ was re-fit only to match the total number of edges in this new reference.

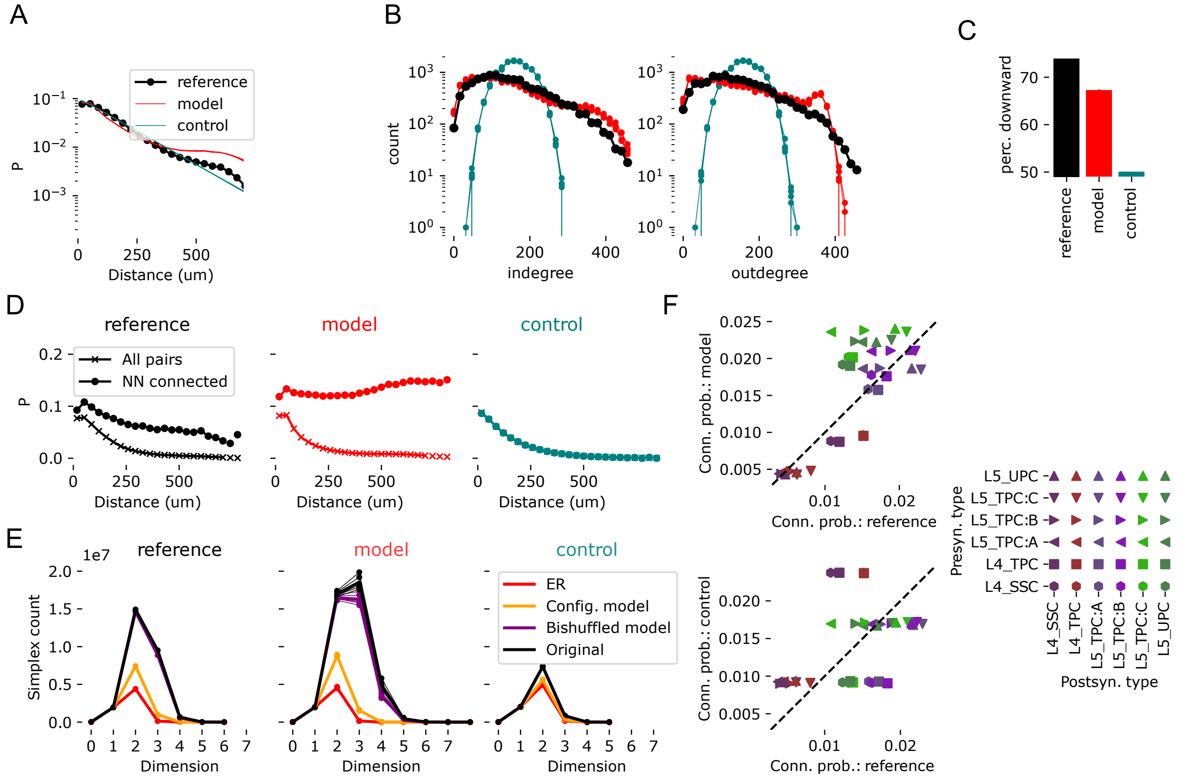


Figure S7: **Modeling connectivity in layers 4/5 of a detailed reference model.** Related to Figure 5. SGSG model compared to excitatory connectivity in layers 4/5 of the rat nbS1 model of Reiman et al. [45] A-E: as in Figure 5, but for the model instead of EM data. F: As in Fig S4.

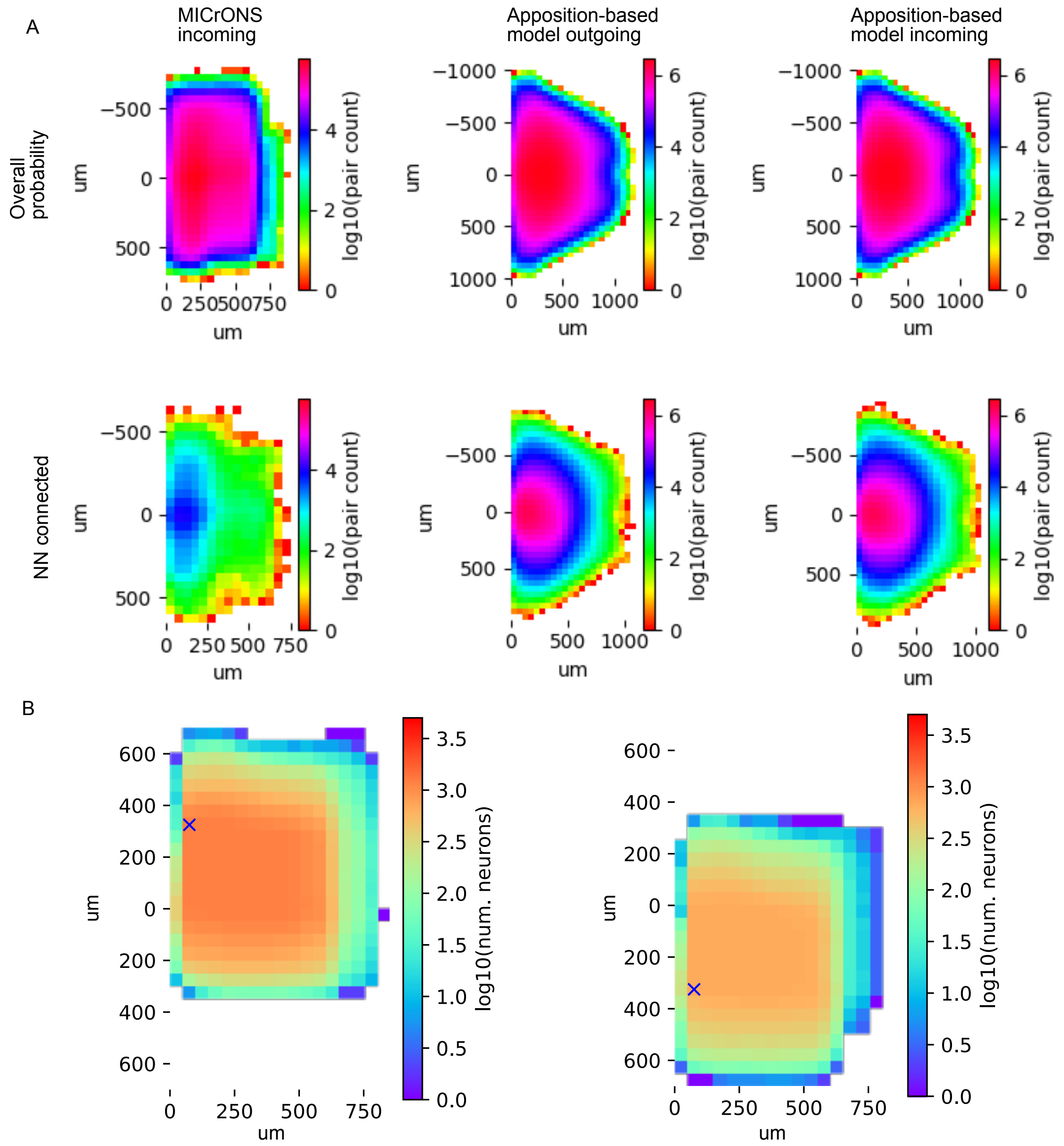


Figure S8: **Number of samples for connection probability estimates.** Related to Figure 3. A: Number of pairs sampled for connection probabilities reported in Fig. 3 that are not already reported in the main figure. B: Number of pairs sampled for the connection probability estimates indicated in Fig. 4A, C and the p-values in Fig 4E.

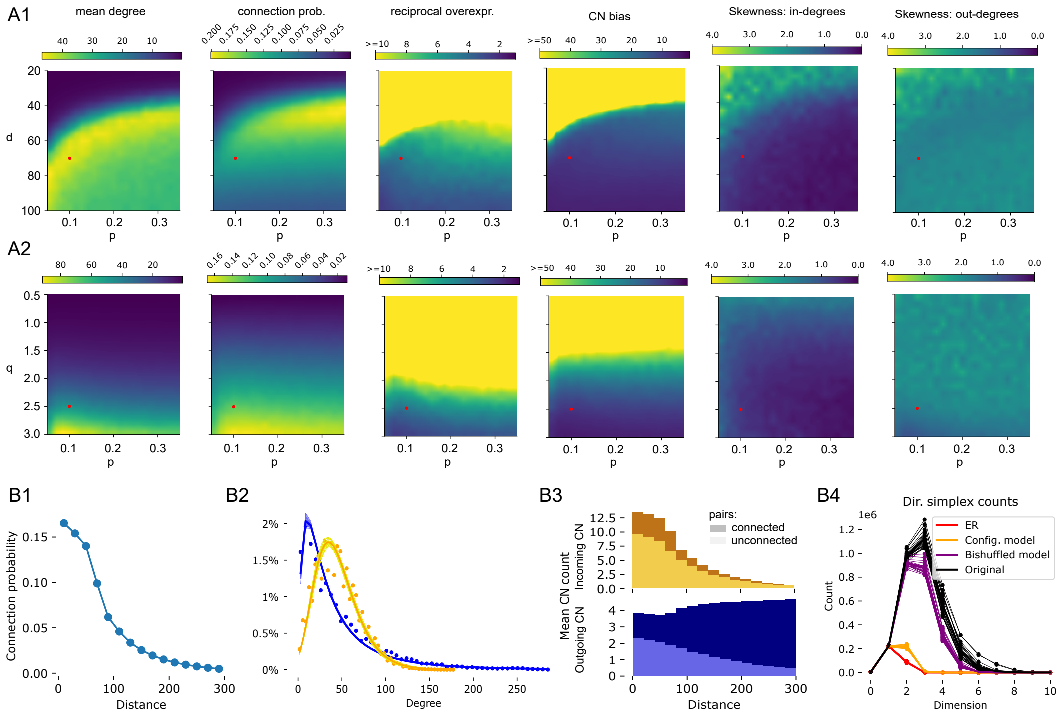


Figure S9: **Parameter scan for the SGSG model.** Related to Figure 5. A1: Values of metrics characterizing overall connectivity and higher-order structure of SGSGs with different values of parameters $p$ and $d$. For explanation of the parameters and metrics, see Methods. A2: Same, but for parameters $p$ and $q$. Red dot indicates location in the parameter space of the instances analyzed further in D. B: More detailed analyses of parameter combination $p = 0.1$, $d = 70\mu m$, $q = 2.5$. B1: Distance-dependence of connection probability. B2: Distribution of in- (orange) and out- (blue) degrees. Dots indicate mean over 25 instances. Thin lines indicate lognormal fits for individual instances, thick line lognormal fit to pooled data. B3: Common neighbor bias: Number of common graph neighbors in SGSG instances for pairs at indicated distances. Light colored: for unconnected pairs, dark: For connected pairs. Top: incoming, bottom: outgoing common neighbors. Mean over 10 instances indicated. B4: Counts of directed simplex motifs in SGSG instances (black) and various controls fit to it. For details on the controls, see Methods. Each line one instance.

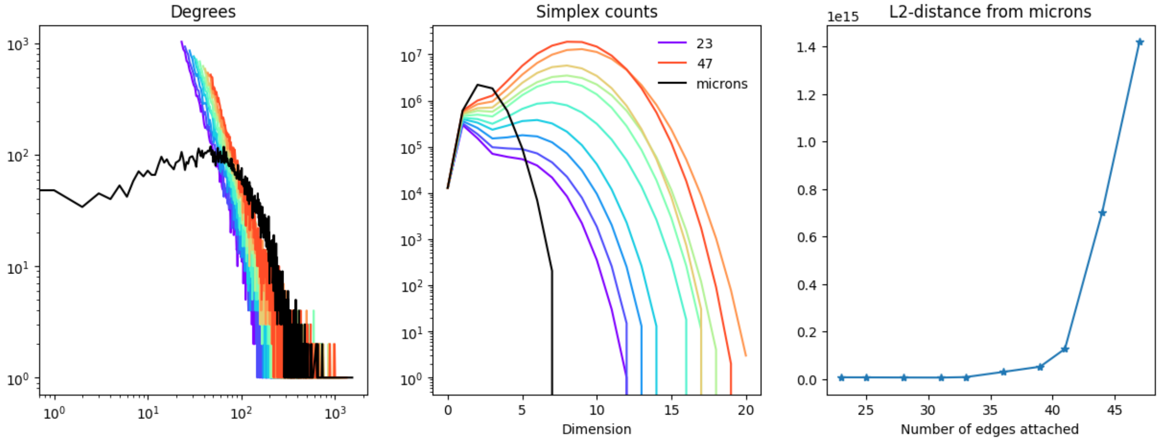


Figure S10: **Fitting the undirected model of Barabási and Albert [29] to the MICrONS data.** Related to STAR Methods. Note that simplex counts in this case are undirected simplices. Different colors indicate different values of the parameter dictating the number of edges (see Methods).

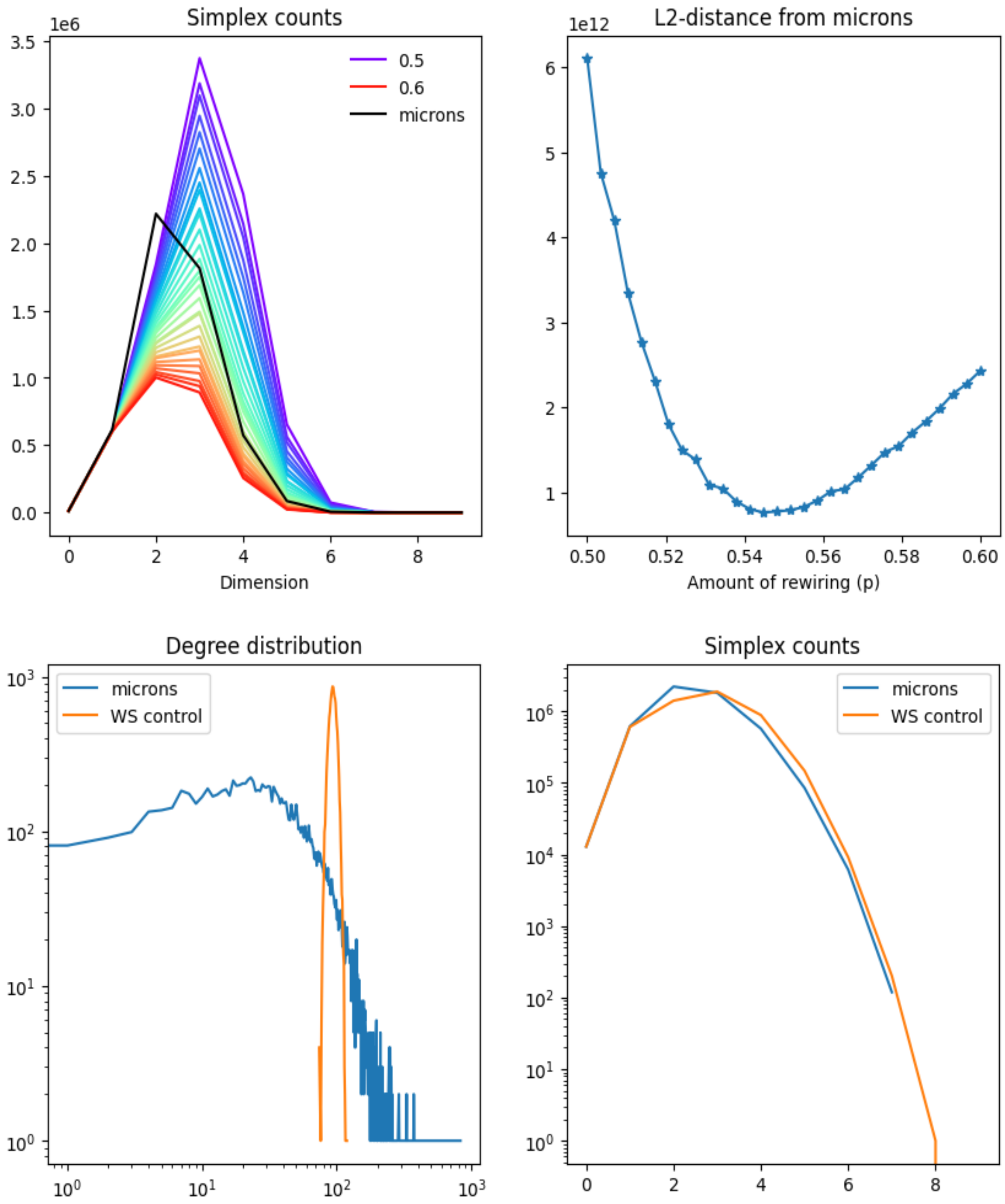


Figure S11: **Fitting a scale-free connectome model [32] to the MICrONS data.** Related to STAR Methods. Top row: Simplex counts and distance of simplex count profile. Bottom right: Simplex counts in logarithmic scale of the best-fitting model. Bottom left: Comparison of degree distributions for the best-fitting model.

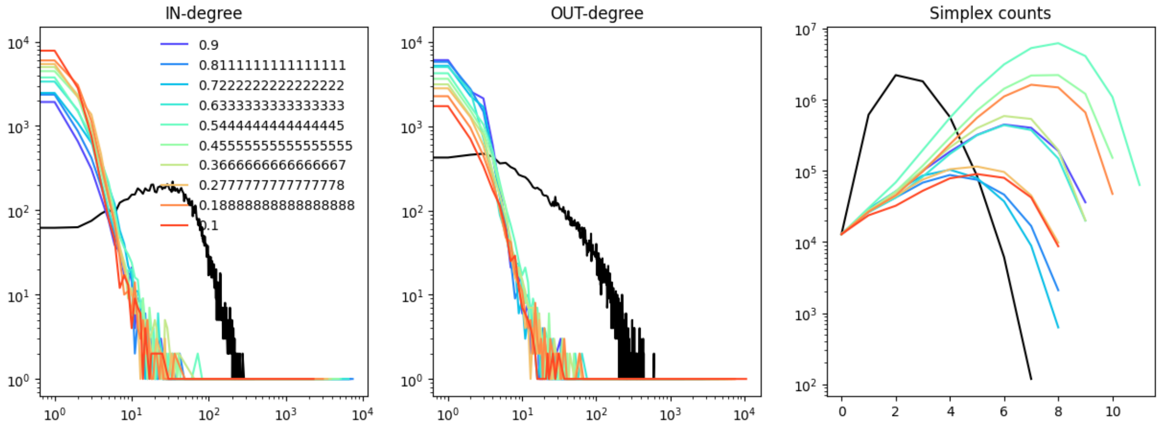


Figure S12: **Fitting the directed preferential-attachment (PA) model of Bollobás et al. [82] to the MICrONS data.** Related to STAR Methods. Left to right: In-degrees, out-degrees and simplex counts. Different colors indicate different parameters for the PA model.

# Methods S1: Mathematical details of the offset-dependent connectivity analysis

### Connection probability, conditional on the nearest neighbor connection

Let $M$ be the adjacency matrix of a network, such as the MICrONS data. That is, $M_{u,v}$ is 1 if there is at least one synaptic contact from neuron $u$ to neuron $v$, and 0 otherwise. We find for each neuron $u$ its *nearest spatial neighbor*, $n(u)$ using a KDTree in the implementation of the "scipy" package in python 3.12. We combined $M$ with the nearest neighbor information to calculate a matrix $N$ such that $N_{u,v}$ is 1 if there is at least one synaptic contact from neuron $u$ to $n(v)$, and 0 otherwise. The overall connection probability is then the mean value of entries of $M$, excluding entries along the diagonal. The outgoing connection probability conditional on the nearest neighbor connected is the mean value of entries of $M$ where the corresponding entry of $N$ is 1, excluding diagonal entries and entries where $n(v) = u$. For the conditional incoming connection probability we simply performed the analysis using the transpose $M^T$ instead. For a distance-dependent analysis we used a matrix of pairwise distances $D$ to identify pairs within a given distance bin; for the two-dimensional version we used two matrices $D_y$ and $D_{x,z}$ denoting the offset along the y-axis and distance in the x,z-plane respectively, as follows.

### Connection probability at an offset

We calculated for each pair of neurons their offsets in the horizontal plane and along the vertical axis. Offsets were then binned in both directions with a bin size of 50 x 50 $\mu m$. Let $B(u, v)$ be the binning function that associates a pair of nodes with a bin. One can think of a bin as a discretized 2D-vector that describes the offset from the position of $u$ to that of $v$. Thus, we refer to a generic bin as $\vec{\Delta}$.

We computed the outgoing and incoming connection probabilities at a given offset over the entire connectome. That is,

$$P^{\rightarrow}_{\vec{\Delta}} = P(u \rightarrow v | B(u, v) = \vec{\Delta}) \qquad \text{and} \qquad P^{\leftarrow}_{\vec{\Delta}} = P(u \leftarrow v | B(u, v) = \vec{\Delta}),$$

where the arrow represents the presence of an edge in the indicated direction and $u$ and $v$ run across all nodes in that offset bin. This is a natural extension of the connection probability at a distance to include vertical direction.

Let $NN(u)$ indicate function that assigns to a node $u$ its *nearest neighbor* according to its spatial location.

We calculated the connection probabilities of each bin, conditioned on the nearest neighbor of a neuron being connected in the given offset. That is,

$$Q^{\rightarrow}_{\vec{\Delta}} = P(u \rightarrow v | B(u, v) = \vec{\Delta}, u \rightarrow NN(v)) \quad \text{and} \quad Q^{\leftarrow}_{\vec{\Delta}} = P(u \leftarrow v | B(u, v) = \vec{\Delta}, u \leftarrow NN(v)).$$

Note in particular, that given our bin size choice, most of the time $u$ and $NN(u)$ belong to the same spatial bin. In this case, $Q^{\rightarrow}_{\vec{\Delta}}$ is the probability of more than one connection existing in the offset bin $\vec{\Delta}$, given that one already exists. Therefore, the difference between $Q^{\rightarrow}_{\vec{\Delta}}$ and $P^{\rightarrow}_{\vec{\Delta}}$ serves as a measure of the degree to which the connectivity in a given bin is statistically non-independent.

### Interdependence of connection probability at different offsets

While the above measured statistical dependencies for a given offset, we also measured statistical dependencies between offsets. That is in some sense, to what extent are $P_{\vec{\Delta}_1}$ and $P_{\vec{\Delta}_2}$ are correlated, for any two of offsets $\vec{\Delta}_1, \vec{\Delta}_2$. To achieve this, we used a more detailed description of the probability structure, defined at the level of individual neurons.

That is, for a fixed neuron $u$ one can calculate its outgoing and incoming connection probabilities at a given offset $\vec{\Delta}$

$$P^{u\rightarrow}_{\vec{\Delta}} = P(u \rightarrow v | B(u, v) = \vec{\Delta}) \qquad \text{and} \qquad P^{u\leftarrow}_{\vec{\Delta}} = P(u \leftarrow v | B(u, v) = \vec{\Delta}).$$

The global connections above, are an average of these values across all neurons $u$. To ensure representative results at the single neuron however, we set the value to be undefined for nodes that had fewer than 100 other nodes at the indicated offset, connected or not.

Let $J(\Delta_1, \Delta_2)$ denote the set of nodes $v$ for which both $P^{u\rightarrow}_{\vec{\Delta}_1}$ and $P^{u\rightarrow}_{\vec{\Delta}_2}$ are defined. That is, those nodes that have at least 100 nodes in both offsets from $v$ independent of connectivity. Then we can build two vectors

$$\mathbf{P}^{J\rightarrow}_{\vec{\Delta}_1} = (P^{u\rightarrow}_{\vec{\Delta}_1})_{v\in J} \qquad \text{and} \qquad \mathbf{P}^{J\rightarrow}_{\vec{\Delta}_2} = (P^{u\rightarrow}_{\vec{\Delta}_2})_{v\in J}.$$

They encode the probability of connectivity for each neuron at each offset. Thus, we calculate their interdependence as the Pearson correlation between them and denote it by $\mathbf{Q}^{\rightarrow}_{(\vec{\Delta}_1,\vec{\Delta}_2)}$. We define $\mathbf{Q}^{\leftarrow}_{(\vec{\Delta}_1,\vec{\Delta}_2)}$ similarly but for incoming connectivity.

To better understand the structure of this measure we clustered the weighted matrices with entries $\mathbf{Q}^{\rightarrow}_{(\vec{\Delta}_1,\vec{\Delta}_2)}$ or $\mathbf{Q}^{\leftarrow}_{(\vec{\Delta}_1,\vec{\Delta}_2)}$ using the Louvain algorithm with a resolution parameter of 1.0. The results were groups of spatial bins that were innervated together by individual neurons (or innervated together individual neurons) more often than expected from the global connection probabilities observed for them.

Additionally, for the plots of Fig. 4C and E, we normalized the connection probability estimates before calculating the correlations. That is, for each neuron we calculated $\tilde{P}^{u\rightarrow}_{\vec{\Delta}} = \frac{P^{u\rightarrow}_{\vec{\Delta}}}{|P^{u\rightarrow}|}$, where $|P^{u\rightarrow}|$ is the mean outgoing connection probability for that neuron across offsets.

# Methods S2: Customizations of SGSGs

### Biasing the spatial neighborhood selection

Above, we constructed a simple random geometric graph by placing connections to nodes in the geometric neighborhood of each vertex $v$, i.e., the set of nodes at euclidean distance smaller than $d$, which we denote by $H_d(v)$, independently at random with probability $p$. We introduce two types of bias into the process. Each bias defines a weight for every edge $(v, w)$, which increases or decreases the probability that the pair is selected. These probabilities are then scaled by their corresponding weights and normalized such that the mean probability over all nodes in $H_d(n)$ remains equal to $p$.

**Per node biases.** The simplest form of bias is to define for each node $v$ a tuple of weights $(w_o(v), w_i(v))$, which increase or decrease the relative out- and in-degree of the node $v$. To do so, we bias the probability of choosing each each edge $(v, u)$ by the weight $w_o(v) \cdot w_i(u)$. While this bias has potentially different values for each node, in practice we used identical values for large groups of nodes, specifically for nodes representing neurons belonging to the same type.

**Orientation-based biases.** This bias is determined by directional alignment with a base unit vector $\overrightarrow{\mathcal{A}} \in \mathrm{R}^n$, which we call the orientation axis. For two nodes $v$ and $u$, corresponding to points $\mathbf{p}_v, \mathbf{p}_u \in \mathbf{P}$, the probability of forming an edge $(v, u)$ is maximally increased when the vector from $\mathbf{p}_v$ to $\mathbf{p}_u$ is parallel and aligned with $\overrightarrow{\mathcal{A}}$; it is maximally decreased when the vector points in the opposite direction, and remains unbiased when it is orthogonal to $\overrightarrow{\mathcal{A}}$.

More precisely, we choose a weight $-1 \leq w_{\mathcal{A}} \leq 1$. Then for any $u \in H_d(v)$ let $\overrightarrow{\mathbf{p}_u - \mathbf{p}_v}$ denote the vector from $v$ to $u$. Then, the bias weight for $(v, u)$ is:

$$w_{\mathcal{A}} \frac{\left(\overrightarrow{\mathbf{p}_u - \mathbf{p}_v} \cdot \overrightarrow{\mathcal{A}}\right)}{\left|\overrightarrow{\mathbf{p}_u - \mathbf{p}_v}\right|} + 1,$$

where $\cdot$ denotes the vector dot product. Note that the bias weight ranges from $1 + w_{\mathcal{A}}$ to $1 - w_{\mathcal{A}}$, depending the level of alignment with $\overrightarrow{\mathcal{A}}$.

### Reducing stochasticity of $S_i(n)$

Above, we describe a process where $S_{i+1}(v)$ is generated from $S_i(v)$ by picking from outgoing neighbors in a random geometric graph independently at random such that the expected size of $S_{i+1}(n)$ is $q$. Here, we introduce an alternative method where instead exactly $\tilde{q}$ candidates are picked, where $\tilde{q}$ is $q$

rounded to the nearest integer. This is similar to the two variants of an Erdos Reyni graph, one with a fixed number of edges and one with a fixed probability of having an edge between a pair of nodes. As there is no variability in the size of $S_i(v)$, we consider this a less stochastic version of the process. We employ the less stochastic version in the first $k$ steps, i.e., for $S_i$ with $i < k$, then switch to the regular version for the remaining steps.

### Extension to long-range connectivity

We extend the SGSG to a proof-of-concept version that incorporates additional long-range connections. To achieve this, we generate two random geometric base graphs over the same set of nodes in 3-dimensional space: one with local connectivity constructed as before, and another where distances are defined by transformed coordinates of the original point could, that reflect the structure of long-range connectivity.

More precisely, from a set of points $\mathbf{P} \in \mathrm{R}^3$, we generate another set of points $\mathbf{P}^{\sim} \in \mathrm{R}^3$ with transformed $y$-coordinates, while keeping the $x$ and $z$ coordinates fixed. The modified $y$-coordinates are given by $\mathbf{P}_y^{\sim} = |\mathbf{P}_y - \mathrm{mean}(\mathbf{P}_y)|$, i.e., the distance from an $x, z$-plane at the center of the point cloud.

Then, we build two random geometric graphs, $G_{d,p}(\mathbf{P})$ and $G_{d,p \cdot m}(\mathbf{P}^{\sim})$ on the same set of nodes, where $m$ is a parameter that determines that balance between the local and long-range edges in the final result. We consider the graph given by the union of edges of $G_{d,p}(\mathbf{P})$ and $G_{d,p \cdot m}(\mathbf{P}^{\sim})$ and denote it by $G^{\mathrm{lr}}_{d,p,m}(\mathbf{P}, \mathbf{P}^{\sim})$. Finally, we build a stochastic spread graph on it, $\mathcal{S}_{q \cdot m}\left(G^{\mathrm{lr}}_{d,p,m}(\mathbf{P}, \mathbf{P}^{\sim})\right)$. Note that other methods to determine $\mathbf{P}^{\sim}$ and versions in which the point cloud is in $n$ dimensional space, are possible and will change the overall and spatial structure of the long-range edges added.
